# Supplementary material for: Are carbon emissions trading and green financial instruments synergistic? -Comprehensive quantitative research based on content analysis
Source: PLoS One. 2024 Mar 7;19(3):e0298601. doi: 10.1371/journal.pone.0298601 (PMC10919601; doi:10.1371/journal.pone.0298601)
Supplement: S1 Data — (ZIP) [file pone.0298601.s001.zip › Data/地级市Z_F工作报告.zip!/╜¡╦╒/╨∞╓▌╩╨/└┤╫╘╣1⁄2╓┌║┼í╛┬φ┐╦╩2╛▌═°í┐_2012.docx]

各位代表：

现在，我代表市人民政府向大会作工作报告，请予审议，并请市政协各位委员和其他列席人员提出意见。

一、2011年工作回顾

2011年是中国共产党建党九十周年，也是“十二五”开局之年。一年来，全市上下在省委、省政府和市委的正确领导下，突出科学发展主题和转变发展方式主线，按照“六个注重”和“两个率先”的新要求，认真实施“八项工程”，扎实抓好“三重一大”，积极推进产业转型，着力改善民生民计，较好地完成了市十四届人大四次会议确定的年度任务，以市为单位在苏北率先基本建成全面小康社会，实现了“十二五”良好开局。

（一）综合实力实现争先进位：地区生产总值突破3000亿元，财政一般预算收入突破300亿元，金融机构新增贷款规模超过300亿元，已有4个县（市）区进入全国百强。

主要指标增幅多数居全省前列。全市地区生产总值预计（下同）完成3600亿元左右、增长13%以上；财政一般预算收入实现318亿元、增长43%。规模以上工业增加值达1750亿元、增长17.5%；服务业增加值达1440亿元、增长14%以上；粮食生产实现“八连增”、总产量突破90亿斤，高效设施农业新增20万亩。在全国、全省金融机构新增贷款规模大幅下降的背景下，我市新增贷款303亿元、增长21%。

三大需求拉动有力。全市固定资产投资完成2200亿元、增长22%，其中工业投资1210亿元；10亿元以上在建项目达58个、增加24个；实际到帐注册外资14亿美元、增长40%，实现两年翻番。社会消费品零售总额1120亿元、增长18%左右，限额以上贸易企业同比增加77%。自营出口37亿美元、增长41%，总量居苏北首位。

县域经济和民营经济增长加快。五县（市）和铜山、贾汪区的地区生产总值和财政一般预算收入增幅分别高于全市1个和8.2个百分点；继铜山、邳州、沛县之后，新沂跨入全国百强县行列，睢宁、丰县综合经济实力明显提升，铜山在苏北率先通过省小康达标验收，沛县、贾汪基本达到小康社会标准。全市民营经济注册资本达2012亿元、增长37%，实现增加值2050亿元、增长15%。

（二）经济建设迈上更高层次：四大主导产业规模全部过千亿，全市百亿元企业达9家，高新技术产业规模突破2000亿元，物联网产业和大学生创业迅猛发展。

主导优势产业向高端攀升。装备制造、食品及农副产品加工和能源产业分别完成产值2068亿元、1612亿元和1000亿元，商贸物流旅游业完成营业收入2190亿元，顺利实现了省委、省政府提出的徐州老工业基地振兴四大千亿元产业发展目标；同时，煤盐化工成为我市又一个千亿元产业。新增5家百亿元企业和3家上市企业，本土上市企业达7家、居苏北首位；徐工集团营业收入超过800亿元，跃居全球同行业第7位，中能硅业多晶硅产能跃居世界第一。现代服务业加快发展，软件开发、服务外包业收入实现当年翻番，67家制造业企业完成主辅分离。

创新型经济增势迅猛。高新技术产业产值增长89%，占规模以上工业比重超过28%、当年提高8个百分点以上。徐州高新区创建取得重要突破；徐州软件园被认定为国家级科技企业孵化器；矿山物联网被列入省十大科技创新工程，两项示范工程通过国家验收，全省唯一的国家级物联网工程实验室落户我市。新建市级以上企业研发机构1432家，总数比上年翻两番，本土大中型企业研发机构实现全覆盖；全市专利授权量达6000件、增长22%。大学生创业和产业化步伐加快，建成5家市级以上大学生创业园，大学生创业企业达1646家。

园区经济实力稳步壮大。国家和省级开发区实现业务总收入5656亿元、增长70%。徐州经济技术开发区综合实力跃居全省前10位，成为省“两化融合”示范基地和低碳经济试点园区；7个省级开发区综合排名大幅提升，特色园区、南北共建园区建设进一步加快。全市新增1家国家级农业产业化示范基地和4家省级现代农业产业园，建成农产品加工集中区15个、数量居全省第一。省市级现代服务业集聚区达50个，集聚服务业企业3727家。

（三）城乡建设扎实推进：率先进入高铁时代，以苏宁、中央百大为重点工程的中心商圈全面开工，30个中心镇创建全面展开，成功获得国家环保模范城市称号。

区域性中心城市建设力度明显加大。年初安排的城建重点工程开工172项、竣工88项，高铁站、三环南路绿化提升、老东门时尚街区等一批精品工程相继完成，主城区、新城区和高铁生态商务区建设有序推进。“大城管”体制和网格化、数字化城管体系逐步健全，市区道路综合整治及重点区域的专项治理效果显著，淮海东路、中山南路被命名为省级市容管理示范路。铜山区和贾汪区加速融入主城区，五个县级城市的功能明显提升；高标准完成30个创建中心镇的规划编制，建成基础设施等项目253个。建设农村公路413公里，完成改厕15万户，解决了51.2万农村人口饮水安全问题，农村水利投资达8.5亿元。淮海经济区核心区一体化建设稳步推进，区域旅游市场开拓、交通联网等9个方面的合作取得积极进展。

城乡生态环境建设全面加强。坚持刚性降耗、铁腕治污，顺利完成淘汰落后产能和污染减排年度任务，单位地区生产总值能耗持续下降，市区空气质量优良天数达335天，国家和省考核断面水质基本达标，市区新增日处理污水能力20万吨、污水管网102公里。大力实施“二次进军荒山”、珠山景区建设等生态工程，大型园林数量达29个，全市森林覆盖率、市区绿化覆盖率分别达31.5%和42%，国家森林城市创建通过考核评估。50个新农村示范村和100个环境综合整治示范村的年度创建任务基本完成。经过坚持不懈的努力，我市成功获得国家环保模范城市称号，呈现出天蓝、水清、地绿的崭新面貌。

（四）和谐社会建设持续强化：农民人均纯收入增幅创1996年以来最好水平，408万平方米的一期棚户区改造工程顺利完成，基本药品价格平均降幅达37.4%，物价房价增幅控制在全省平均水平以下。

民生民计明显改善。以“六大体系”建设为抓手，在强力推进年度民生实事工程建设的同时，精心安排并启动实施了总投资110亿元的9大类19个民生幸福工程项目计划。“两增两控两保”实现预期目标，城镇居民可支配收入和农民人均纯收入分别达到19200元和9500元，增长14.5%和18%以上，年纯收入低于2500元的农村人口基本实现脱贫目标，城镇新增就业10万人，登记失业率控制在3%以内；采取综合措施稳定物价、控制房价，及时落实特殊群体临时价格补贴，重点抓好11家农贸市场和70家平价商店建设，主动实施市区商品房限购政策和价格备案制度，全市CPI涨幅在全省平均水平以下，普通商品住房价格涨幅已连续9个月下降；城镇居民医保、新农合、新农保、城乡低保基本实现应保尽保，城镇居民社会养老保险付诸实施；保障性住房建设超额完成全年任务，3.5万平方米农村偏远学校教师公租房建成使用，380余万平方米的二期棚户区改造工程正式启动。

各项社会事业统筹推进。教育均衡化发展和现代化创建步伐加快，各级各类教育水平稳步提升，98%以上的中小学实现标准化办学，加固和重建校舍52.7万平方米，创建省优质幼儿园60所、省四星级学校5所、国家中等职业教育改革发展示范学校3所，徐州幼师升格为高等专科学校。基层医疗卫生机构综合改革扎实开展，基本药物制度实现全覆盖，完成10大类公共卫生服务项目年度任务。文化行政管理机构改革全面完成，县级经营性文化事业单位及文艺院团转企改制加快实施，文化产业发展加快，城乡文明程度明显提高，我市荣获全国文明城市提名。人口计生综合改革不断深化，人口出生强度得到初步遏制。残疾人“五个一”帮扶工程全面完成，镇级工作网络实现全覆盖。全民健身实施计划深入推进，奥体中心开工建设，省十八届运动会承办工作顺利开展。双拥模范城“七连冠”创建成效突出，军地合作共建再上新台阶；高质量完成“慰烈工程”各项任务，为全国烈士褒扬工作创造了新经验。人防、民兵、民族宗教、档案、外事、侨务、台湾事务、统计、审计、广播影视、新闻出版、住房公积金管理、无线电管理、淮塔管理、机关事务管理、防震减灾、气象、妇女儿童、青少年、关心下一代、老龄、慈善、援疆等各项事业都取得了新进展。

和谐稳定的社会局面持续巩固。“法治徐州”、“平安徐州”建设深入推进，社会稳定风险评估、矛盾纠纷排查调处及社会治安防控等机制不断完善，法律援助覆盖面明显提高。严格落实领导干部接访、带案下访和包案制度，大力开展专题调研、“百日百案”和“三解三促”活动，有效解决了一批事关群众切身利益的突出问题。大力强化安全生产和食品药品安全工作，严格落实安全生产责任制，着力加强重点领域的隐患排查和安全监管，进一步完善应急管理体系，有力维护了安全稳定的社会大局。

（五）政府自身建设取得新的成效：科学发展分类考核深入推进，争先进位氛围更加浓厚，行政权力网上公开透明运行办件量居全省前列，行政复议和行政诉讼案件数量明显下降。

机关作风和效能建设持续深化，市级机关绩效考核、县（市）区综合考核和镇级分类考核的引导作用不断强化，各地各部门奋力争先进位、推动跨越发展的主动性显著提升。政府权力运行的内控机制进一步完善，“12345”公共服务热线和声频报网“四位一体”行风热线建成开通。积极用创新的思路破解资金、土地等要素制约，银企对接和融资会办制度深入实施，国土资源管理共同责任制全面落实。全市上下合力攻坚，事关改革发展稳定全局的20件大事顺利推进，徐台航线开通运营，军用机场搬迁等取得重要进展。廉政建设深入推进，公务用车等专项治理取得阶段性成果，预防和惩治腐败工作力度不断加大。自觉接受市人大及其常委会的法律和工作监督，主动接受市政协及社会各界的民主监督，政府法制建设继续加强，依法行政水平进一步提升。

各位代表，过去的一年，我市经济社会发展的成绩来之不易，这是全市973万人民凝心聚力、锐意进取、奋力拼搏的结果。在此，我代表市人民政府，向全市各族人民群众、向人民解放军驻徐部队、武警官兵和公安民警，向各民主党派、工商联、无党派人士和人民团体，向支持徐州现代化建设的香港特别行政区同胞、澳门特别行政区同胞、台湾同胞、海外侨胞和各界人士，向投身徐州经济社会发展的国内外投资者和企业家，表示衷心的感谢！

在肯定成绩的同时，我们也清醒地看到，当前经济社会发展还存在不少困难和问题。主要是：生产要素供应趋紧，企业生产成本明显上升，部分企业特别是中小企业经营困难加大；服务业占比偏小，现代服务业的规模和层次亟待提升；产业总体结构依然偏重，节能减排形势严峻；物价房价上涨压力依然较大，保持城乡居民收入持续较快增长的难度增加，交通拥堵问题较为严重，改善民生、创新社会管理和加强安全生产监管的任务仍很艰巨；政府工作还存在薄弱环节，效率效能建设仍需进一步强化。对此，我们将高度重视，采取切实有效措施加以解决。

二、2012年工作总体部署

今年是实施“十二五”规划承上启下的重要一年，也是深入贯彻省市党代会精神、迎接党的十八大胜利召开的重要一年，做好今年工作意义重大。今年政府工作的基本思路是：坚持以邓小平理论和“三个代表”重要思想为指导，深入贯彻落实科学发展观，牢牢把握主题主线，坚持稳中求进、好中求快，全面落实“八项工程”，全力推进“三重一大”，着力转变经济发展方式，着力增进民生幸福，着力建设区域性中心城市，着力深化改革开放，又好又快推进“两个率先”，不断开创全市科学发展新局面。

今年经济社会发展的主要预期目标是：（1）地区生产总值增长13%左右；（2）财政一般预算收入增长18%；（3）固定资产投资增长22%；（4）社会消费品零售总额增长18%左右；（5）实际到帐注册外资增长15%；（6）自营出口总额增长15%；（7）城乡居民收入实际增长12%以上；（8）居民消费价格涨幅低于全省平均水平；（9）城镇登记失业率控制在4%以内；（10）节能减排完成省定任务；（11）人口自然增长率控制在6‰以内。

做好今年的工作，有利条件较多，困难挑战也不少。世界经济复苏的不稳定性不确定性上升，经济下行风险加大；国家确定了稳中求进的总基调，国内经济继续朝着宏观调控预期方向发展；我市经济发展的基础日益扎实，增长内生动力持续增强。我们将密切关注宏观形势变化和政策导向，紧密结合徐州实际，积极抢抓机遇，主动破解难题，大力扶持实体经济发展，按照科学发展的要求，更好地实现跨越式增长、内涵式增长、自主式增长、绿色增长、协调增长和包容性增长的有机统一。在工作推进过程中，将切实强化以下五个方面：一是更加突出争先进位。强力推动各县（市）区跨越发展，着力培育和放大新的竞争优势，努力实现速度指标争先、总量指标进位、结构指标提升、人均指标前移，确保地区生产总值突破4000亿元、财政一般预算收入突破400亿元、新增信贷规模保持在300亿元以上。二是更加突出转型升级。加大产业结构战略性调整力度，深入实施科技创新工程，确保高新技术产业产值超过3000亿元、全市百亿元企业达11家、本土上市企业达10家以上。三是更加突出扩大内需。把经济增长的主动力放在扩大内需上，进一步优化投资结构、扩大消费需求，同时着力优化出口产品结构，确保固定资产投资突破2600亿元、社会消费品零售总额突破1300亿元、出口总额突破45亿美元。四是更加突出节能减排。不断强化低碳绿色的增长理念，确保全面完成省下达的节能减排任务，组织开展PM2.5监测试点，启动建设30个中心镇污水处理厂，全市新增绿化造林8万亩。五是更加突出民生改善。扎实推进民生幸福工程建设，深入实施“居民收入六年倍增计划”，持续加强基层基础建设，提升社会管理的科学化水平，确保城镇居民可支配收入和农民人均纯收入分别突破2.1万元和1万元，实现城乡居民社会养老保险全覆盖，新建改建100所农村义务教育学校，着力抓好70项为民办实事项目。

三、更大力度推进经济又好又快发展

坚持保增长与促转型有机结合，稳步提高发展的全面性、协调性和可持续性。

（一）以推进“三重一大”为重点，着力优化经济增长的动力结构。

强力推进“三重一大”。集中力量实施总投资3219亿元的200项重大项目建设，其中当年实施170项、年度投资795亿元。扎实推进城市轨道交通、亿吨大港、南水北调、湖西航道整治等20项基础设施项目建设，积极抓好徐工装载机智能化升级扩建、中能多晶硅四期、维维运动饮料等54项“六大千亿元工业产业”项目，加快实施中心商圈、水上乐园及配套设施、雨润农副产品全球采购中心、龙湖湿地旅游景区、徐州云计算数据中心等80项现代服务业和新兴产业项目，统筹推进6项社会事业项目等，积极做好徐工重型汽车等30项前期项目的推进工作，为保增长调结构提供有力支撑。

积极拓展消费空间。进一步增强消费对经济增长的拉动力，继续提升中心商圈服务功能，加快特色商业街区建设，推进家居装潢、汽车汽配、农副产品等专业市场提档升级，着力打造区域性消费中心。加快镇村标准化农家店建设，推动农村商业连锁经营和统一配送，充分挖掘农村消费潜力。注重培育新的消费热点，大力发展电子商务、居家养老、家庭服务等新型业态。坚决打击商业欺诈、制假售假行为，营造安全放心的消费环境。

稳步提升外经贸水平。继续巩固亚洲、欧盟、北美等传统市场，主动开拓东盟、拉美等新兴市场。强化对进出口企业的帮扶，支持徐工、中能、艾德等企业完善国际营销体系，帮助更多企业申报出口经营权。稳定工程机械、光伏、板材、大蒜等重点商品出口，提升品牌和高新技术产品的出口比重，加快发展服务贸易，提高加工贸易水平，努力创建国家级出口基地和贾汪保税物流中心。鼓励企业开展境外投资、建立海外研发机构和生产加工基地。

（二）以构建现代产业体系为重点，着力加大转型升级力度。

不断强化农业现代化建设。积极开展粮食高产创建，加快农田水利和高标准农田建设，推进农业科技进步，增强农业综合生产和抵御风险能力。深入实施农业提档升级行动计划，重点抓好现代农业“1+5”工程，力争年内新增设施农业20万亩、大中型规模养殖场20个，努力提升绿色农业、有机农业的比重。创新农业和农村经营体制，加快发展农民专业合作组织，完善农产品流通体系，加强农产品质量管理和品牌创建，加大国家和省级农业龙头企业培育力度，提高农产品的附加值和市场竞争力。

高水平推进新型工业化。围绕打造“六大千亿元工业产业”，重点培育壮大特色装备制造业板块，加快推进传统能源产业升级和新能源产业链条延伸，继续提升食品及农副产品精深加工水平，加大煤盐化工、冶金、建材产业技术改造力度，力争六大产业规模达到8000亿元左右。强化大企业大集团培育，力争徐工集团营业收入达到千亿元，新增2家百亿元工业企业；强化上市企业梯队建设，新增3家上市企业。加快创建省级信息化和工业化融合示范基地，努力新增20家“两化融合”示范企业。

加速构建现代服务业高地。坚持现代服务业加快发展和传统服务业巩固提升并重，重点打造商贸、物流、金融、房地产、旅游“五大千亿元服务业产业”。引导鼓楼、云龙、泉山区分别重点培育2-3个特色服务业产业，促进各县（市）和铜山、贾汪区做大服务业总量，力争全市服务业增加值占比提高1个百分点。重点培育5家左右超50亿元企业（市场），继续实施制造业企业主辅分离，推进软件开发、服务外包业倍增发展，科技服务、商务服务等产业大幅增长。把文化产业作为现代服务业的重要内容，精心编制“十二五”文化产业发展规划，大力发展文化旅游、数字文化、演艺娱乐、文艺服务、出版印刷、文博展销等产业，加快淮海艺术品交易中心、国家级动漫影视基地和龟山博物馆景区建设，打响“舞动汉风”品牌。

强力推动创新型经济跨越发展。强化科技政策落实、高新技术企业培育和区域创新试点创建，深入推进企业研发机构建设，确保规模以上工业企业覆盖率达85%，高新技术和新兴产业产值均增长80%以上。深入推进大学生创业，提升创业园和产业化基地承载能力，力争新增1000家大学生创业企业。推动矿山物联网成套技术攻关和产业化发展，拓展医疗物联网等其他领域，力争物联网产业产值达700亿元左右。加大国家级高新区创建力度，有序推动技术孵化和产业集聚，早日建成苏北及淮海经济区科技创新高地和成果转化基地。高水平建设中国矿大大学科技园，推动徐师大文化创意产业园创建国家级大学科技园，规划建设中美(徐州)创新科技示范园，积极构建多元投入的科技金融服务体系，建立300家校企联盟。

有效破解发展瓶颈制约。落实最严格的耕地保护制度，规范实施增减挂钩和万顷良田建设工程试点，用好采煤塌陷地、工矿废弃地、闲置地等存量土地，大力推进多层标准厂房建设，积极整合农村小散居住点，提高节约集约用地水平，严肃查处整改违法违规用地行为。围绕建设区域性金融中心，继续加大招金引银力度，大力引进股份制商业银行和创业投资、风险投资等各类基金，积极利用直接融资工具拓展融资渠道，切实加强对民间借贷行为的监管，严厉打击非法集资。引导企业加强同职业技术院校的合作，组织开展订单、定向式培训，缓解用工结构性紧缺的矛盾；加强企业发展研究院、创新创业教育学院等人才基地建设，建成国家级淮海人力资源交流中心，加大招才引智力度，不断强化人才支撑。

（三）以构筑“八大中心”为重点，着力抓好“美好城乡”建设。

加快提升中心城市的现代化水平。围绕区域性“八大中心”建设目标，精心修编城市总体规划和各类专项规划，科学引领城市发展布局。大力实施道路通畅、环境整治等7大类城建重点工程建设。重点推进老城区三环东路高架快速路、新淮海西路、韩山隧道等道路畅通工程，高标准建设珠山景区、三环北路等景观绿化项目；重点实施新城区昆仑大道、汉源大道和环大龙湖“三条轴线”的连片开发，突出教育卫生等公共服务项目建设；重点抓好高铁生态商务区长途汽车站、公交首末站等功能性项目建设；统筹抓好铜山区、贾汪区的规划建设，加快融入主城区的步伐。开展省级优秀管理城市、市容管理示范路和幸福家园示范小区创建，完善数字化城市管理系统，加大主次干道、居民小区及背街小巷的综合整治力度。推动公交优先发展，加快道路渠化和路网建设改造，加强停车场（点）规划建设，强化交通组织疏导，努力解决交通拥堵问题。积极推进淮海经济区核心区合作发展，继续加强区域旅游市场开拓、科技成果交流等方面的深层次合作。

统筹推进县级城市和中心镇建设。进一步完善现代城镇体系，支持各县（市）加快提升城市功能，强化30个中心镇创建工作。加快推进基础设施、公共服务设施向镇村延伸，加大垃圾收运、水源清洁、园林绿化等环境保护和生态建设力度，年内建设农村公路120公里、桥梁100座，力争区域供水覆盖面达到55%，完成2498个村庄的环境整治任务，争创15个三星级“康居乡村”，创建50个新农村达标示范村。

（四）以创新体制机制为重点，着力深化改革和扩大开放。

抓好重点领域和关键环节的改革。继续深化行政审批制度改革，完成事业单位岗位设置管理和绩效工资改革，实施经济发达镇行政管理体制改革试点。着力抓好财税体制改革，建立预算绩效管理新模式，做好结构性减税相关工作。加快农村信用社改革，完成丰县、沛县、铜山和贾汪区农信社改制组行任务，培育更多的面向小微型企业和“三农”的金融机构。稳妥推进农村各项改革。进一步深化医药卫生体制改革。

加快提升民营经济的发展层次。认真落实促进民营经济发展的政策意见，鼓励民间资本更多地进入现代服务业、新兴产业等领域。巩固完善中小企业金融顾问和银企对接制度，争取更大力度的信贷支持，强化对小微型企业的政策帮扶。加强对100家重点成长型企业的培育指导，支持中小企业和大企业大集团开展配套合作，加速向集群集聚方向发展。

努力提高招商活动的质量和实效。突出主导产业、新兴产业、现代服务业等项目的招商，抓好项目包装推介和前期洽谈，精心组织3项综合性招商活动和14项专题招商活动，强化签约项目的跟踪落实。鼓励各地、各有关部门大力组织特色招商和专题招商活动。积极推动外资企业开展增资扩股，引导资金投向产业链高端、研发和营销等环节。

进一步增强园区经济的发展活力。推动徐州经济技术开发区加速壮大高端工程机械、光伏光电两大支柱产业，加快建设机器人特色产业基地、总部经济园等专业园区，积极创建国家级生态工业园区。引导7家省级开发区加强特色园区建设，分别重点培育2-3个优势产业，加快壮大丰县电动车、沛县煤盐化工、睢宁白色家电、邳州循环经济、新沂新医药等主导产业，采取有效措施提升44个工业集中区发展水平。继续深化南北共建，力争所有开发区均建立南北共建园区。抓好中央商务区、创意产业园等7类现代服务业集聚区建设。高水平建设铜山国家级现代农业示范区、沛县国家级农业产业化示范基地，加快推进省级现代农业产业园区、农产品加工集中区提档升级。

（五）以强化节能减排为重点，着力抓好生态文明建设。

坚决打好节能减排攻坚战。严格落实目标责任，推行节能减排风险抵押金制度，实行合同能源管理，控制“两高”和产能过剩项目上马，确保40%以上的年耗能3000吨标准煤以上企业实施节能改造。大力抓好污染治理，强化现有污水处理厂运营监管和提标改造，完善配套管网，加快实施30个中心镇污水处理厂建设，推进电力、钢铁行业脱硫以及水泥行业脱硝工程，减少农业面源污染。

全面加快生态市创建。扎实推进生态市和生态园林城市建设，实现国家森林城市创建目标。深入开展“二次进军荒山”计划，抓好城乡绿化和采煤塌陷地生态修复，加快中德合作示范区建设。开展PM2.5监测试点，强化对机动车尾气、建筑扬尘和粉煤灰等方面治理，确保空气环境质量优良天数超过90%。加快实施丰县、沛县、睢宁、新沂尾水资源化和导流工程等34项淮河流域治污项目，巩固9个断面保护区建设成果，确保水质稳定达标。加快主城区内化工企业退城入园和转型升级步伐，加强重金属污染和涉铅企业的治理整顿，完成34个国家级生态镇、54个省级生态镇和517个市级以上生态村创建任务。

四、更加扎实推进民生改善和社会管理创新

深入推进民生幸福工程，认真落实民生实事项目，全面提升民生“六大体系”建设水平。

（一）把增加群众收入作为最大实事，深入实施城乡居民收入倍增计划。全面落实强农惠农政策，加强农村劳动力技能培训和创业扶持，更大力度抓好新一轮扶贫开发，促进农民收入较快增长。稳步提高企业最低工资标准和工资指导线，积极推进职工工资集体协商，完善职工工资正常增长机制，不断扩大中等收入者比重。健全完善社会救助体系，强化对失业人员、“三无人员”、残疾人等困难群体的帮扶，进一步提升城乡低保补贴标准。切实强化物价房价监管，抓好重点农产品的成本控制和生产供应，加快平价商店及农贸市场建设，落实好困难群体价格上涨补贴制度，确保居民生活质量和水平稳步提升。

（二）把强化民生保障作为基本目标，加快构建“六大体系”。按照“分类指导、突出重点、典型带动”的思路，深入开展大学生集群创业、农村偏远学校教师公租房建设、优质幼教资源加快向农村延伸、平价农贸市场建设等典型示范，带动整体工作有序开展。在终身教育体系方面，继续加大教育投入，提升各类学校的办学质量，年内创建省市级优质幼儿园80所，新建改建100所农村义务教育学校，力争邳州市通过省教育现代化评估验收；扎实推进“校舍安全”、“校园安全”工程，加固重建中小学校舍85万平方米；全面落实职业培训普惠制补贴政策，确保年内新增职业技能培训4万人。在就业服务体系方面，大力推动自主创业，支持劳动密集型和小微型企业发展，深入实施农村公共就业服务“四项制度”，年内新增城镇就业8万人。在社会保障体系方面，进一步提高各类社会保障的覆盖范围、保障标准和统筹层次，重点推进城乡居民社会养老保险全覆盖，努力实现被征地农民社会保障“即征即保”。在基本医疗卫生体系方面，加大卫生资源整合力度，重点抓好第一人民医院迁建、中心医院新城区分院、徐医附院东院和精神病院建设，完成27个城市社区卫生服务中心的升级改造，积极创建示范乡镇卫生院和标准化卫生室。在住房保障体系方面，重点抓好保障性住房建设管理，确保全面完成省定任务，组织建设1044套农村偏远学校教师公租房，积极推进二期棚户区改造，继续实施商品房价格备案制度和限购政策。在社会养老服务体系方面，启动建设200个居家养老服务中心、一批公办老年公寓和养老机构，继续实施农村敬老院“关爱工程”，加快构建多元化养老服务设施建设格局。同时，着力抓好10家农贸市场建设和12万户农村改厕等70项为民办实事项目，尽力解决群众反映集中的突出问题。

（三）把完善公共服务作为重要任务，统筹推进其他各项社会事业发展。高度重视文化事业发展，深入实施文化惠民工程，抓好市民活动中心、未成年人教育基地建设，建成10个街道和100个社区文化中心，构建覆盖城乡的公共文化服务体系；加强精神文明建设，大力实施文明徐州人形象塑造计划，强化文艺精品创作和文化遗产保护，积极创建全国文明城市。做好第18届省运会筹备和备战训练工作，力争奥体中心主体建设完工，加快城市社区和行政村健身点建设。切实稳定低生育水平，降低出生人口性别比，创建国家人口计生综合改革示范市。实施残疾儿童抢救性康复等工程，建设市残疾人康复中心。巩固双拥模范城创建成果，落实好优抚安置政策，促进双拥工作制度化、经常化、社会化。统筹抓好人防、民兵、民族宗教、档案、外事、侨务、台湾事务、统计、审计、广播影视、新闻出版、住房公积金管理、无线电管理、淮塔管理、机关事务管理、防震减灾、气象、妇女儿童、青少年、关心下一代、老龄、慈善、援疆等方面的工作。

（四）把维护和谐稳定作为第一责任，不断提升社会管理科学化水平。健全完善立体化的社会治安防控体系和网格化的社会矛盾调处体系，着力化解城市建设管理、工资及劳动保障等重点领域的信访矛盾，加强社会治安监控系统建设，力争年内所有县（市）区以及90%的镇、村达到综治规范建设标准。深入开展和谐社区建设，确保全市65%的社区、40%的行政村建成和谐社区（村）。严格落实安全生产责任制和“三同时”制度，采取综合措施强化校车安全专项治理，加强食品药品安全的监管和服务，积极推行安全生产责任保险，扎实开展煤矿、交通等重点行业领域的安全检查，实施安全隐患挂牌督办制度，有效防范和坚决遏制重特大事故发生。

各位代表，今年的经济社会发展任务繁重而艰巨。为了确保各项任务全面落实、各项目标顺利实现，我们将以政府换届为契机，不断加强政府自身建设。坚持不懈地推进创新型政府建设，大力弘扬“三创三先”精神，突出围绕“八项工程”和“三重一大”，进一步提升政府的执行力和效率效能，对确定的目标、制定的政策、部署的工作一抓到底。坚持不懈地推进法治政府建设，把依法办事贯穿于政府工作各个环节，切实规范行政执法行为，严格依照法定权限和程序行使权力、履行职责。坚持不懈地推进服务型政府建设，着力改进机关工作作风，不断完善“12345”热线等政府服务平台，增强服务基层、服务企业、服务群众的针对性。坚持不懈地推进廉洁政府建设，严格执行党风廉政建设责任制，建立健全勤廉评价体系，坚决查办违纪违法案件，强化厉行节约，主动压缩行政开支，始终保持清正廉洁的政府形象。坚持不懈地推进透明政府建设，主动接受市人大及其常委会的法律和工作监督、市政协的民主监督，积极支持统一战线工作，充分发挥各民主党派的作用，自觉接受社会公众监督和新闻舆论监督，促进领导干部养成在监督环境下工作的习惯，保证权力在阳光下运行。

各位代表，新的形势、新的任务对我们提出了新的更高的要求。让我们紧密团结在以胡锦涛同志为总书记的党中央周围，全面贯彻落实省委、省政府和市委的决策部署，抢抓机遇，攻坚克难，乘势而上，确保圆满完成全年各项目标任务，以更加优异的成绩向党的十八大献礼！
